# Role of actin-binding proteins in prostate cancer

**DOI:** 10.3389/fcell.2024.1430386

**Published:** 2024-07-11

**Authors:** Fangzhi Fu, Yunfeng Yu, Bo Zou, Yan Long, Litong Wu, Jubo Yin, Qing Zhou

**Affiliations:** Department of Andrology, The First Affiliated Hospital of Hunan University of Chinese Medicine, Changsha, China

**Keywords:** prostate cancer, actin, actin-binding protein, actin filaments, cytoskeleton

## Abstract

The molecular mechanisms driving the onset and metastasis of prostate cancer remain poorly understood. Actin, under the control of actin-binding proteins (ABPs), plays a crucial role in shaping the cellular cytoskeleton, which in turn supports the morphological alterations in normal cells, as well as the invasive spread of tumor cells. Previous research indicates that ABPs of various types serve distinct functions, and any disruptions in their activities could predispose individuals to prostate cancer. These ABPs are intricately implicated in the initiation and advancement of prostate cancer through a complex array of intracellular processes, such as severing, linking, nucleating, inducing branching, assembling, facilitating actin filament elongation, terminating elongation, and promoting actin molecule aggregation. As such, this review synthesizes existing literature on several ABPs linked to prostate cancer, including cofilin, filamin A, and fascin, with the aim of shedding light on the molecular mechanisms through which ABPs influence prostate cancer development and identifying potential therapeutic targets. Ultimately, this comprehensive examination seeks to contribute to the understanding and management of prostate diseases.

## 1 Introduction

Prostate cancer (PCa) stands as one of the most prevalent malignancies affecting men globally, holding a prominent position among male malignant tumors on a worldwide scale. In light of the anticipated rise in the elderly population, PCa is poised to become a pivotal concern in disease prevention and management (Miller et al., 2022). The defining features of PCa primarily involve resistance to androgen deprivation and the propensity for metastasis. Research indicates that an estimated one-third of patients will ultimately transition to castration-resistant prostate cancer (CRPC) (Coleman et al., 2020; Fares et al., 2020). A significant majority of CRPC cases culminate in bone metastasis, a pivotal factor that influences both the quality of life and the survival rates of PCa patients (Zhang et al., 2021). The metastatic cascade of tumor cells encompasses dynamic transformations in the cellular cytoskeleton, where reshaping is instrumental in fueling invasion and metastasis, reflecting alterations in cell morphology, migration capabilities, cytokinesis, and phagocytic activity (Xu et al., 2023). Comprised of actin filaments, microtubules, and intermediate filaments, the cytoskeleton has emerged as a focal point in recent investigations demonstrating how anticancer medications can remodel cytoskeletal components to confer therapeutic benefits (Ahmad et al., 2023).

**FIGURE 1:**
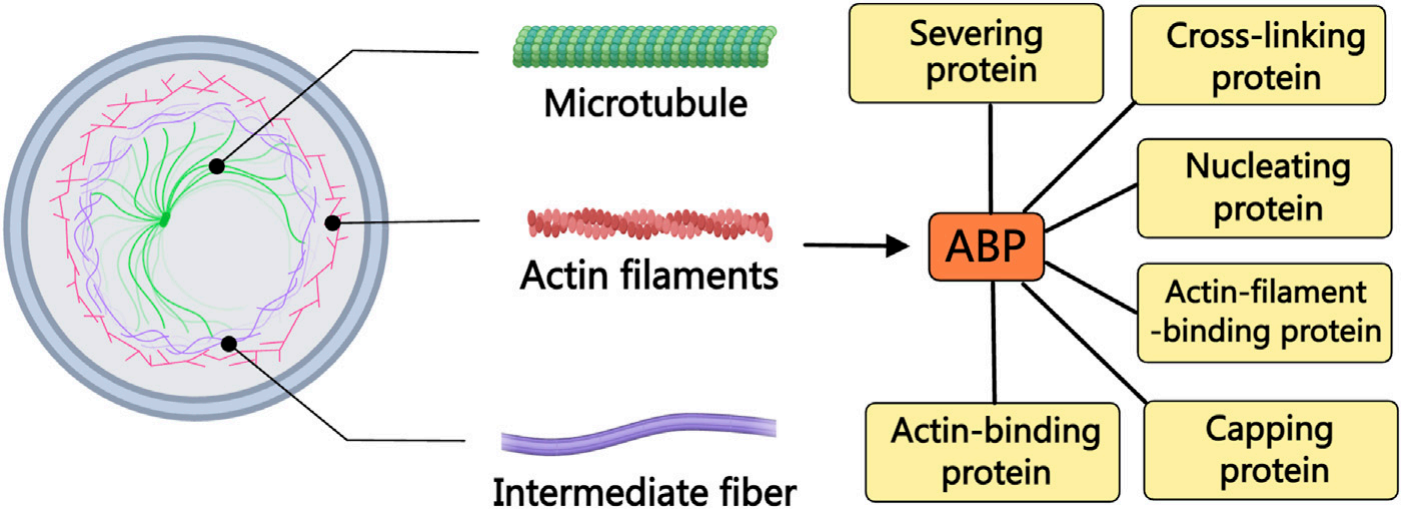
The relationship between ABPs and the cytoskeleton. The cytoskeleton is composed of actin filaments, microtubules, and intermediate filaments. The dynamics of actin filaments are regulated by various ABPs. Based on the differences in regulating various aspects of actin assembly, ABPs can be divided into different types of proteins, including ABPs, actin filament-binding proteins, severing proteins, nucleating proteins, capping proteins, and cross-linking proteins.

Actin, a predominant constituent within the cellular cytoskeleton, plays indispensable roles in processes like cellular shape modulation, division, and migration (Merino et al., 2020). Nonetheless, the dynamics of actin are finely tuned by a repertoire of actin-binding proteins (ABPs) (Gao and Nakamura, 2022). Operating as key regulators, ABPs oversee actin’s involvement in nucleation, elongation, and disassembly processes, effectively bridging the cellular membrane and the nucleus (Kadzik et al., 2020). Since their initial characterization in the 1970s, over 160 distinct ABPs have been cataloged (Zhang et al., 2021). Illustratively depicted in Figure 1, ABPs can be categorized into several types based on their roles in modulating different facets of actin assembly, encompassing ABPs, actin filament-binding proteins, severing proteins, nucleating proteins, capping proteins, and cross-linking proteins. With diverse functionalities, various ABPs facilitate the aggregation of numerous actin molecules, orchestrate the formation of new filaments, facilitate filament elongation, cap filament ends to curb elongation, sever filaments, and interlink filaments (Vakhrusheva et al., 2022). Recent literature highlights the involvement of ABPs in the initiation and migration of PCa through the regulation of oncogene expression, exemplified by cofilin, the Arp2/3 complex, TPM1, FlnA, and TAGLN (Izdebska et al., 2020; Kmeťová Sivoňová et al., 2021). Building upon these insights, this review succinctly surveys the recent advancements in our understanding of various ABPs associated with PCa, offering novel avenues for combatting and mitigating the progression of this disease.

## 2 Actin filament severing in PCa

Cofilin, a 19 kDa actin filament-severing protein widely present in eukaryotic cells (Shishkin et al., 2017), is a key member of the ADF/cofilin family, which encompasses ADF (actin-depolymerizing factor), cofilin-1, and cofilin-2, pivotal in regulating the dynamics of cellular actin filaments (Maciver and Hussey, 2002). While ADF and cofilin-1 exhibit widespread expression across various non-muscle tissues, cofilin-2 is predominantly found in muscle tissues like the heart and prostate, with cofilin-1 being the subject of more comprehensive studies (Bamburg and Wiggan, 2002). Notably, cofilin-1 emerges as a promising pathological biomarker in PCa tissues, offering insights into tumor progression, metastasis, and treatment outcomes (Lu et al., 2015; Stark et al., 2017; Kiełb et al., 2023; Sousa-Squiavinato and Morgado-Díaz, 2024). Shishkin found an upregulation of mRNA and protein levels of cofilin-1 in PCa cells compared to non-malignant cells (Shishkin et al., 2017). Additionally, differential protein expression analyses in PCa cell lines with varying metastatic abilities highlight cofilin-1 as a significantly distinct protein, suggesting its vital role in cancer invasion and metastasis (Chen et al., 2020). Enhanced filopodium formation and cell migration capabilities in cofilin-overexpressing PCa cells further underscore the pivotal involvement of cofilin-1 in tumor progression (Collazo et al., 2014). Recent investigation elucidates the role of cofilin-1 in driving cancer migration through asymmetric actin polymerization, thereby influencing the cell motility cycle and extracellular matrix interactions (Warner et al., 2024), hinting at the intricate mechanisms by which cofilin regulates actin filaments to impact PCa advancement.

Various regulatory mechanisms modulate the activity of cofilin-1, including phosphorylation/dephosphorylation, phosphatidylinositol 4, 5-bisphosphate binding, and intracellular pH regulation (Bravo-Cordero et al., 2013; Sousa-Squiavinato and Morgado-Díaz, 2024). Particularly in PCa cells, the phosphorylation/dephosphorylation of cofilin-1 at serine 3 (Ser3) is a predominant event. Phosphorylation of cofilin Ser3, primarily catalyzed by LIM domain kinases (LIMK1 and LIMK2) under normal conditions (Mizuno, 2013), leads to the inactivation of cofilin, thereby stabilizing the actin cytoskeleton (J. Park et al., 2021). LIMK1/2-mediated phosphorylation of cofilin emerges as a critical therapeutic target in PCa management, inhibiting cofilin severing activity, cytoskeletal reorganization, filopodia formation, and chromosomal abnormalities to impede PCa progression at early stages (Davila et al., 2003; 2007; Shah and Cook, 2023). Given the pivotal role of the androgen receptor (AR) in PCa occurrence and progression, LIMK inhibitors show promise in reducing PCa cell motility, diminishing AR protein stability and transcriptional activity, thereby holding therapeutic potential in PCa treatment (Mardilovich et al., 2015).

**FIGURE 2:**
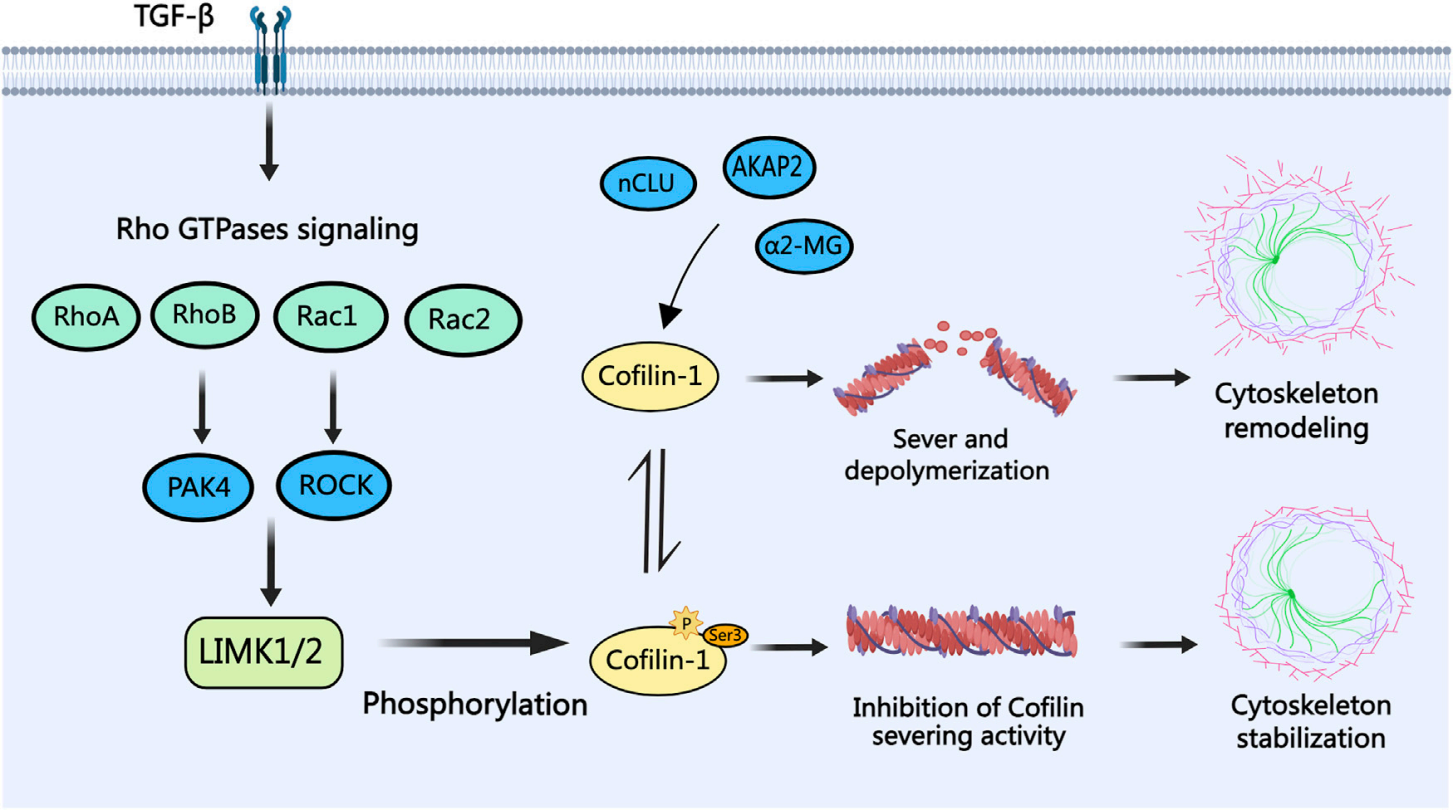
The role of cofilin-1 in PCa. Transforming growth factor-beta (TGF-β) modulates Rho family GTPases (RhoA, RhoB, Rac1, and Rac2) signaling, leading to cofilin-1 phosphorylation via LIMK1/2. Phosphorylated cofilin-1 loses its severing activity, ensuring actin cytoskeleton stability. Conversely, activation of cofilin-1 by proteins like α2-MG, nCLU, and AKAP2 through dephosphorylation facilitates actin filament severing and depolymerization, culminating in cytoskeletal reorganization, filopodia formation, and consequent migration and invasion of PCa cells.

In PCa cells, as depicted in Figure 2, cofilin-1 functions as a downstream effector of Rho family GTPases signaling (B. Chen et al., 2019). Rho family GTPases, including RhoA, RhoB, Rac1, and Rac2, elicit tumor-suppressive effects by activating PAK4, ROCK, or directly stimulating the LIMK/cofilin signaling pathway (Ahmed et al., 2008; Alfano et al., 2012; Lee et al., 2019; X; Zhang et al., 2019). Notably, within the context of cancer epithelial-mesenchymal transition (EMT), Transforming Growth Factor-β (TGF-β) assumes a pivotal role in modulating cell cytoskeleton alterations via the activation of Rho GTPases signaling pathways. Investigations have indicated cofilin, as a downstream target of TGF-β, drives the TGF-β-induced invasion and metastasis of PCa cells (Zhu et al., 2006; Collazo et al., 2014). Conversely, alpha-2-macroglobulin (α2-MG), nuclear clusterin (nCLU), and anchoring protein AKAP2 exert reverse control over cofilin dephosphorylation and activation, thereby influencing the dynamics of actin filaments to promote PCa cell invasion and metastasis (Misra et al., 2005; Moretti et al., 2011; Reggi et al., 2024). Given these findings, the modulation of cofilin phosphorylation to regulate actin cytoskeleton dynamics in cancer cells emerges as a promising avenue for potential therapeutic intervention in PCa.

Cofilin-1 plays a crucial role in the pharmacological mechanisms of various therapeutic drugs for PCa. For instance, docetaxel activates the cofilin-1/Paxillin signaling pathway, prompting apoptosis in PCa by suppressing cofilin-1 expression (Xiao et al., 2016). Pérez-Martínez utilized nanodelivery technology to administer a specific siRNA targeting cofilin-1 to human PCa cells, downregulating cofilin-1 expression and enhancing docetaxel-induced cytotoxicity (Pérez-Martínez et al., 2012). In another study, Lampe employed nanodelivery technology to administer the drug cabazitaxel, resulting in increased expression of phosphorylated cofilin at Ser3, impacting cancer cell migration and invasion pathways (Lampe et al., 2023). Thus, chemotherapy drugs can exhibit anticancer effects by inhibiting cofilin-1 expression or deactivating phosphorylated cofilin-1. Resistance to PCa treatment may also be linked with cofilin-1, as revealed in a proteomic study comparing docetaxel-sensitive and -resistant PCa cells by Zu et al., identifying cofilin as a key differential protein in PCa cell migration (Zu et al., 2015). Enzalutamide, a widely used AR inhibitor, is associated with drug resistance in advanced treatment stages. Paller’s study on the combination therapy of the TGF-β receptor I inhibitor galunisertib and the antiandrogen enzalutamide demonstrated significant reduction in cofilin expression levels, thereby optimizing the therapeutic response to enzalutamide (Paller et al., 2019). Investigations on azithromycin resistance mechanisms unveiled that cofilin-1 triggers the P38 MAPK signaling pathway, facilitating actin filament cytoskeletal reorganization and elevating the expression and drug efflux activity of multidrug resistance protein 1, consequently compromising the efficacy of PCa treatment (Chen et al., 2020). Active components derived from natural plants inhibit cell viability and induce apoptosis in PCa cells mediated through cofilin-1. For example, ursolic acid, a traditional Chinese medicine component, induces apoptosis in PCa cells by targeting the ROCK/PTEN-mediated cofilin-1 mitochondrial translocation (Gai et al., 2016; Mu et al., 2018). Huperzine E, a key component in traditional Chinese medicine, triggers apoptosis in human PCa cells by engaging cofilin-1 and mTORC1 pathways (X. He et al., 2017). In contrast, Huperzine F enhances cofilin-1 phosphorylation, impeding PCa cell proliferation through the promotion of actin aggregation and cofilin-actin rod formation (Ren et al., 2012). The compound curcumin suppresses cell motility by diminishing protein kinase D1 downstream target activity and elevating cofilin phosphorylation levels (Sundram et al., 2012). Subsequent to treatment with thapsigargin, there is a notable rise in cofilin-1 protein expression in PCa cells, hindering tumor growth by modulation of the F-actin/cofilin-1/paxillin pathway and the Akt-mTOR pathway (Huang et al., 2018).

Tumor suppressor genes and oncogenes play pivotal roles in the initiation and advancement of PCa. Alterations or inactivation of these genes can precipitate tumor growth and metastasis. HSPB6, a significant tumor suppressor gene, carries prognostic implications; decreased expression in PCa is often associated with a bleak outlook. Feng et al. investigated therapeutic activators and identified 8-Br-cGMP as capable of activating HSPB6 and inducing dephosphorylation of phosphorylated Cofilin, eliciting apoptosis in PCa cells. Notably, the synergistic effect of 8-Br-cGMP and quinidine notably bolstered HSPB6 transcription levels, effectively restraining PCa growth and offering promising prospects for PCa treatment (Feng et al., 2024). Overexpression of miR-608 targets RAC2/PAK4/LIMK1/cofilin, thereby impeding PCa progression (X. Zhang et al., 2019). Similarly, upregulation of the tumor suppressor gene miR-143 thwarts the LIMK1/cofilin signaling pathway, reinstating arsenic-induced PCa apoptosis (Ngalame et al., 2016). Oncogenic Circ-FOXO3 boosts the viability, motility, and proliferation of PCa cells through the miR-1299/cofilin-2 axis, while suppressing apoptosis (P. Li et al., 2021). Furthermore, the oncogene lncRNA-SOX2 enhances cell proliferation and migration in PCa via the miR-369–3p/cofilin-2 axis (Wo et al., 2019). Overexpression of the oncogene ERG in prostate epithelium spurs cofilin activity, fostering EMT that fuels prostate tumor genesis (Griner et al., 2015). Additionally, the oncogene SOX11 exhibits heightened transcriptional activation in metastatic PCa tumors, elevating cofilin activity and cell migratory capabilities (Hirokawa et al., 2020). Thus, cofilin emerges as a crucial downstream effector protein influenced by an array of tumor suppressor genes and oncogenes, regulating the cellular cytoskeleton by modulating actin filaments, governing cell proliferation, apoptosis, and migration, and playing a pivotal role in the evolution and progression of PCa. An array of therapeutic strategies targeting cofilin is detailed in Table 1.

**TABLE 1:** Therapeutic strategies by regulating cofilin.

Therapeutic strategies	Model	Main observations	Refs
Docetaxel inhibited the expression of cofilin-1	LNCaP cells	Cell growth was inhibited, paxillin expression was decreased, apoptosis was increased, and caspase-3 activity was increased	Xiao et al. (2016)
Cabazitaxel nanoparticles promote cofilin-1 phosphorylation	PC 3 and C4-2B cells	Vimentin expression was decreased, E-cadherin expression was increased, p-120 catenin expression was decreased, and IL-8 expression was decreased	Lampe et al. (2023)
cofilin-1 siRNA combined with docetaxel inhibited the expression of cofilin-1	LNCaP cells	Increased caspase-3 activity, increased apoptosis, cell cycle arrest and inhibition of cell proliferation were observed	Pérez-Martínez et al. (2012)
TGF-β receptor I inhibitor combined with Enzalutamide inhibited cofilin-1 expression	Transgenic Mouse Model of PCa (TRAMP DNTGFβRII)	Cell growth was inhibited, apoptosis was increased, and Smad 4 was significantly downregulated, inducing the MET phenotype transformation	Paller et al. (2019)
Ursolic acid inhibited cofilin-1 expression	LNCaP, DU 145 cells	Cell proliferation was inhibited, cell apoptosis was increased, Caspase-3/9 activity was increased, PTEN and cytochrome c protein expression was increased, and ROCK expression was decreased	Gai et al. (2016), Mu et al. (2018)
Cucurbitacin E inhibited cofilin-1 expression	LNCaP cells	Cell apoptosis was increased, and the expression of mTOR protein, AMPK protein, p53 protein and caspase-9 was increased	(X. He et al., 2017)
Cucurbitacin F promotes cofilin-1 phosphorylation	DU145, PC 3, LNCaP cells	cofilin-actin rod formation, cell cycle arrest at G (2)/M phase, increased p21 protein expression and decreased cyclin A expression	Ren et al. (2012)
Curcumin promoted cofilin-1 phosphorylation	C4-2, PC 3, and LNCaP cells, tumor xenograft mice	Cell proliferation was inhibited, protein kinase D1 expression was increased, nuclear β-catenin transcriptional activity was inhibited, membrane β-catenin expression was increased, and tumor growth was inhibited	Sundram et al. (2012)
Thapsigargin promoted cofilin-1 expression	PC 3 cells	Cell proliferation was inhibited, cell apoptosis was increased, Caspase-3/9 activity was increased, and the expression of p-Akt protein, mTOR and paxillin was decreased	Huang et al. (2018)
8-Br-cGMP inhibited cofilin phosphorylation	LNCaP, VCaP, C4-2, DU 145, PC 3, 22Rv 1 cells	HSPB 6 expression was increased, E2F1 expression was decreased, and apoptosis was increased	Feng et al. (2024)

## 3 Actin filament cross-linking in PCa

### 3.1 Filamin (FlnA)

**FIGURE 3:**
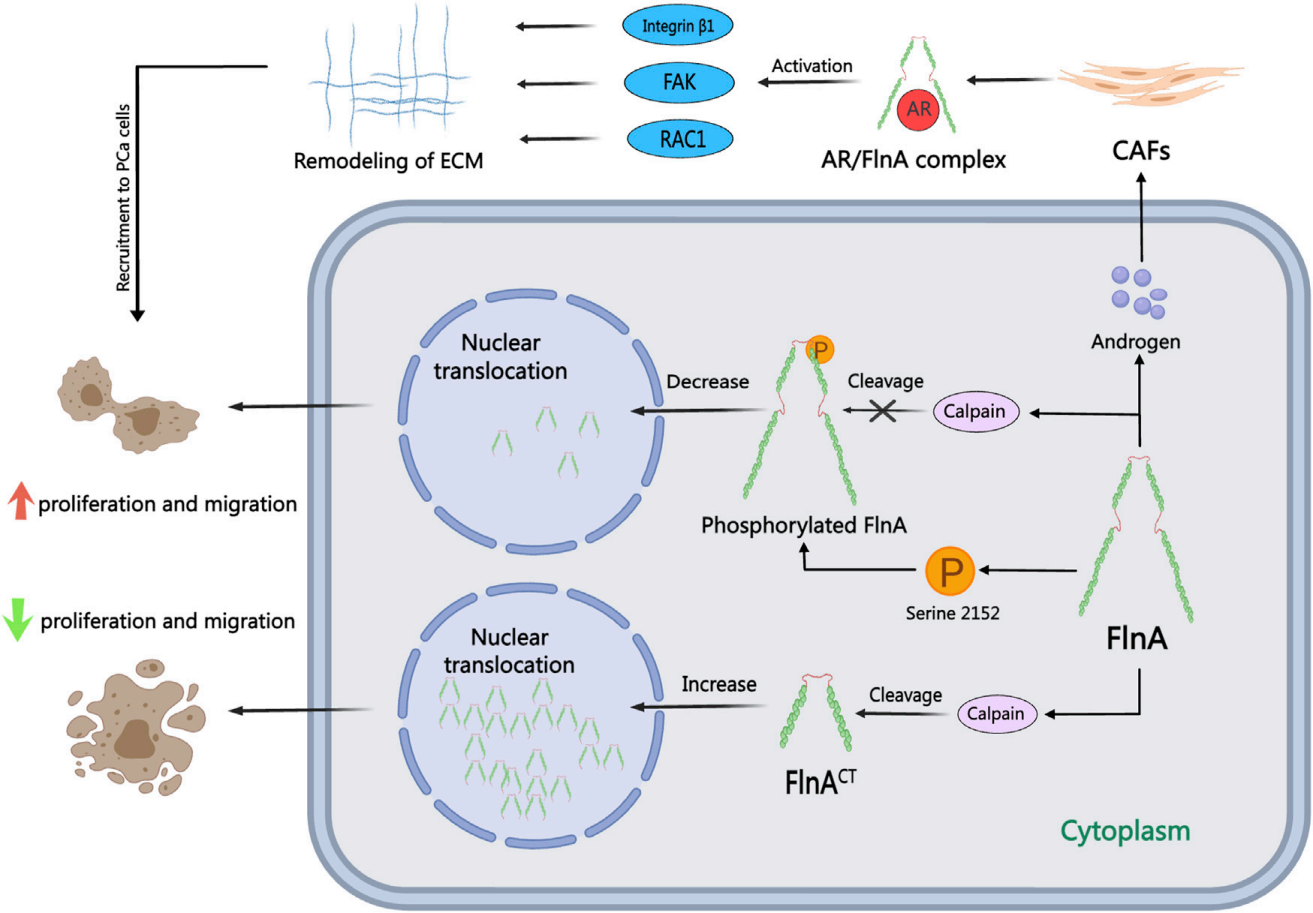
The role of FlnA in PCa. FlnA can undergo calpain-induced cleavage to produce a 90 kDa carboxy-terminal fragment (FlnA^CT^), which translocates to the nucleus via cellular signaling cascades. In the cytoplasm, FlnA promotes tumor proliferation and migration, while nuclear localization inhibits these processes. In the presence of androgens, FlnA forms an AR/FlnA/integrin β1 complex activating Rac1, FAK, and MMP-2 pathways, driving extracellular matrix remodeling and enhancing the invasion capability of CAFs.

FlnA, a 280 kDa actin-crosslinking protein, acts as a pivotal nuclear transcription regulator directly influencing AR functionality in the nucleus, contributing to the pathogenesis and progression of PCa (Loy et al., 2003). Recent investigations suggest that serum FlnA levels can serve as an innovative biomarker for stratified PCa screening, surpassing PSA in screening efficacy (Panigrahi et al., 2019; Leitão et al., 2022; Mahaveer Chand et al., 2024). Functionality of FlnA in PCa cells varies based on its cellular localization, as illustrated in Figure 3: while cytoplasmic localization of FlnA fosters tumor growth and migration, nuclear localization hampers tumor progression and migration (Shao et al., 2016). The role of calpain is crucial for the nuclear translocation of FlnA and subsequent AR activation. Normally, calpain cleaves FlnA to generate a 90 kDa carboxy-terminal fragment (FlnA^CT^), which navigates to the nucleus through cell signaling pathways and regulates transcription factors. In AR-negative cells, calpain inhibitors safeguard FlnA and the AR ligand-binding domain from cleavage (T. Liu et al., 2014). In a study conducted by Salimi, inhibition of calpain-mediated FlnA cleavage resulted in reduced production of FlnA^CT^, subsequently diminishing the proliferation, migration, and colony-forming capabilities of PCa cells (Salimi et al., 2018). Additionally, McGrath confirmed that calpain-mediated cleavage of FlnA drives the nuclear localization of the AR transcriptional coactivator FHL2. FHL2 activation sustains AR activity in CRPC, thereby impeding the effectiveness of Enzalutamide treatment in CRPC cases (McGrath et al., 2013). Consequently, combining calpain inhibitors with ADT may emerge as a viable treatment strategy to impede or postpone the progression of PCa.

FlnA, a pivotal AR protein partner, plays a significant role in the metastasis and dissemination of PCa in response to androgens (Castoria et al., 2017). The notable loss of FlnA’s nuclear localization is a hallmark of CRPC, primarily attributed to FlnA phosphorylation, which hampers cleavage and nuclear translocation. This alteration disrupts the transcriptional profile orchestrated by AR, yet augmentation of nuclear FlnA expression can reestablish androgen sensitivity in CRPC cells (Savoy et al., 2015). In investigations on resistance to antiandrogen medications like bicalutamide, Wang induced the re-entry of FlnA into the nucleus, leading to diminished Akt phosphorylation. Nuclear FlnA expression can impede the proliferation of androgen-independent cells, thus reinstating sensitivity to bicalutamide treatment. Protein Kinase A (PKA) inhibitors have the capacity to obstruct FlnA phosphorylation at Ser 2,152, thereby impeding FlnA cleavage and its nuclear translocation (Wang et al., 2007). Moreover, PKA inhibitors can suppress AR transcriptional activity and impair cell growth in low-androgen environments (Bedolla et al., 2009). The combination of the natural product genistein with polysaccharides induces FlnA cleavage and nuclear localization, inhibiting FlnA phosphorylation and consequently sensitizing PCa cells to ADT (Mooso et al., 2012). AR/FlnA complexes can be utilized to gauge susceptibility and track disease progression in PCa (Zhang et al., 2023). Stimulated by androgens, the AR/FlnA complex in cancer-associated fibroblasts (CAFs) recruits integrin β1 and activates Rac1 and FAK, thereby facilitating the migration of PCa cells (Castoria et al., 2011). Di Donato has pioneered the development of AR-derived peptides in laboratory settings that can selectively neutralize the effect of androgens on PCa cells and CAFs. These peptides inhibit PCa cell invasion by disrupting the assembly of the AR/FlnA/integrin β1 complex and activating downstream Rac1, FAK, and MMP-2 signaling pathways (Di Donato et al., 2015; Di Donato et al., 2021a; Di Donato et al., 2021b). This targeted intracellular approach against the AR/FlnA complex in PCa-associated fibroblasts demonstrates enhanced efficacy in overcoming resistance to second-generation inhibitors in PCa.

Moreover, FlnA contributes to the pathogenesis and advancement of PCa through a myriad of mechanisms beyond its connection with the AR. In their study, Cahuzac et al. identified that the PARP inhibitor olaparib targets pre-activated autophagy, resulting in diminished SQSTM1/p62 nuclear localization and an elevation in FlnA expression, enhancing treatment flexibility (Cahuzac et al., 2022). Enhanced levels of Transforming Acidic Coiled-Coil Protein 3 (TACC3) disrupt the interaction between FlnA and meckelin, impeding primary cilia formation in PCa cells (Qie et al., 2020). The tumor-suppressive role of the TGF-β-mediated FlnA/R-Smad signaling pathway is observed in normal prostate epithelial cells (S. Assinder and Cole, 2011). Additionally, the long non-coding RNA LINC01002 demonstrates potential anti-cancer efficacy in PCa by targeting the miR-650/FlnA pathway (Qian et al., 2023). While the involvement of FlnA in modulating the aforementioned molecular pathways is acknowledged, its precise mechanisms of action remain incompletely understood. Future investigations should thoroughly explore these pathways to advance the development of therapeutic strategies focusing on FlnA regulation.

### 3.2 Fascin-1 (FSCN1)

FSCN1, part of the Fascin family of ABPs, possesses a molecular weight of approximately 55 kDa (H. Liu et al., 2021). This protein is extensively studied in cancer cell metastasis due to its role in cross-linking actin filaments into densely packed parallel structures (Z. Li et al., 2022). Neuroendocrine prostate cancer (NEPC), a high-grade subtype of PCa, exclusively exhibits FSCN1 expression (Turpin et al., 2023), underlining its association with increased invasiveness, metastasis, and decreased patient survival rates (Darnel et al., 2009; Zhang et al., 2023). Overexpression of FSCN1 in PCa cells triggers the generation of invasive pseudopodia, fostering cell invasion, migration, and EMT (Sarantelli et al., 2023), making FSCN1 a compelling therapeutic target for PCa.

**FIGURE 4:**
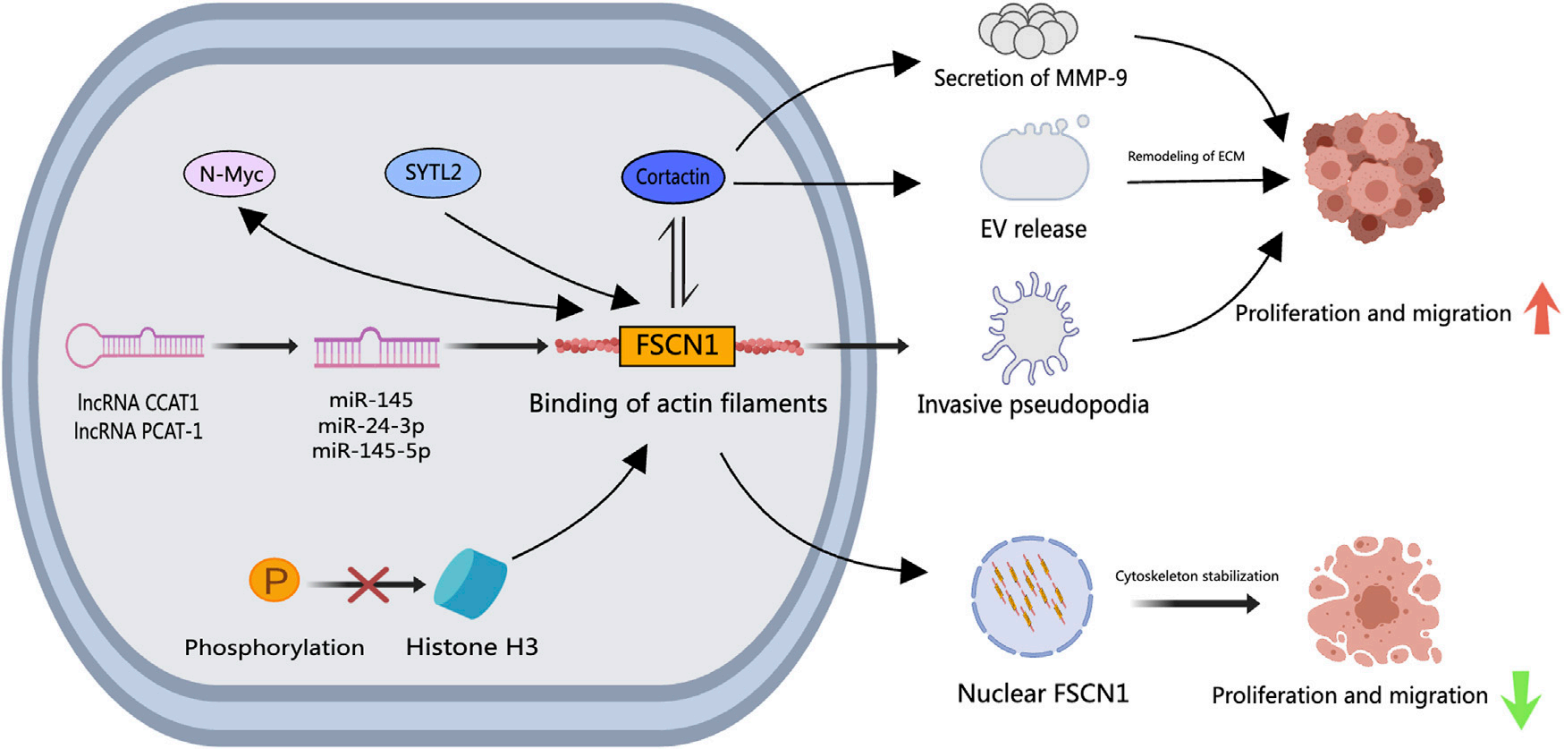
The role of FSCN1 in PCa. Upstream signaling factors such as N-Myc, SYTL2, CCAT1, and PCAT-1 enhance FSCN1 expression in PCa cells, where a bidirectional regulatory mechanism between N-Myc and FSCN1 exists. FSCN1 facilitates the formation of invasive podosomes, promoting PCa cell invasion and metastasis. Together with Cortactin, FSCN1 regulates the release of Extracellular Vesicles and MMP-9 secretion, inducing extracellular matrix remodeling that facilitates PCa cell invasion and metastasis. Inhibition of histone H3 results in nuclear FSCN1 accumulation and actin filament assembly, exerting repressive effects on PCa cell invasion and metastasis.

Studies have outlined FSCN1’s involvement in PCa progression through diverse cellular signaling pathways. The actin-binding protein Cortactin collaborates with FSCN1, modulating extracellular vesicle (EV) release, pseudopodia formation, MMP-9 secretion, and related processes (Van Audenhove et al., 2014; Beghein et al., 2018). Synaptotagmin-like 2 (SYTL2) is intertwined with high metastasis rates, advanced tumor staging, and poor prognosis in PCa. SYTL2 upregulation impedes the proteasomal degradation of FSCN1 and enhances PCa cell mobility by regulating pseudopodia formation. The SYTL2-FSCN1-pseudopodia axis holds promise as a therapeutic target for metastatic PCa (Z. Li et al., 2023). N-Myc proto-oncogene protein (N-Myc) escalation has been linked to the malignant advancement of PCa. An intricate interplay exists between N-Myc and FSCN1, where N-Myc potentiates FSCN1 expression and, conversely, FSCN1 can suppress N-Myc expression (G. He et al., 2020). Furthermore, miR-145, miR-24–3p, and miR-145–5p, downregulated in PCa, act as tumor suppressors by directly modulating FSCN1 (Fuse et al., 2011). The oncogenic long non-coding RNA colon cancer-associated transcript 1 (CCAT1), upregulated in PCa, regulates miR-24–3p expression. Knockdown of CCAT1 enhances paclitaxel sensitivity in PCa by modulating the miR-24–3p/FSCN1 pathway (X. Li et al., 2020). Similarly, the long non-coding RNA PCa-associated transcript 1 (PCAT-1) contributes to PCa by reducing miR-145–5p expression, promoting cell proliferation, migration, invasion, and hindering apoptosis through FSCN1 activation (Weibo et al., 2017). Histone H3 serves a crucial role in the temporal and spatial regulation of nuclear FSCN1. Nuclear FSCN1 exerts anti-cancer effects through actin bundling for chromatin organization and efficient DNA repair (Lawson et al., 2022). Inhibiting histone H3 phosphorylation, causing nuclear FSCN1 accumulation and actin filament assembly, may explain the anti-cancer properties of nuclear FSCN1. As shown in Figure 4, these collective findings unravel varied signaling mechanisms through which FSCN1 influences PCa metastasis and progression. Nevertheless, targeted therapeutic drugs specifically for FSCN1 are lacking, paving the way for future development of multi-targeted drugs to concurrently inhibit diverse FSCN1-associated signaling pathways. Notably, the small molecule inhibitor NP-G2-044 targeting FSCN1 has shown efficacy in ocular neovascularization (Bai et al., 2023), hinting at its potential in PCa as it inhibits cell migration and filopodia formation.

### 3.3 Ezrin

Ezrin, a member of the Ezrin-radixin-moesin (ERM) protein family, functions as a crucial link between the plasma membrane and actin cytoskeleton, profoundly influencing cancer progression and metastasis (Clucas and Valderrama, 2014; Kawaguchi and Asano, 2022). Highly prevalent in PCa cells and circulating tumor cells (CTCs), Ezrin serves as a valuable biomarker for tumor metastasis and prognostication (Z. Chen et al., 2022; Valdman et al., 2005). Research indicates that Ezrin governs the migratory and invasive capabilities of PCa cells through phosphorylation (Chuan et al., 2006). In the presence of androgens, Ezrin phosphorylation triggers c-Myc oncogene overexpression. This increased c-Myc protein synthesis and degradation inhibition confer an invasive phenotype upon PCa cells, with downstream signaling pathways involving Akt and GSK-3β (Chuan et al., 2010). Notably, the anti-adhesive transmembrane sialomucin Podocalyxin forms a complex with Ezrin, where Podocalyxin expression correlates with elevated Ezrin phosphorylation. The Podocalyxin/Ezrin complex induces heightened MMP expression, enhanced MAPK and PI3K activity, culminating in a more aggressive PCa phenotype (Sizemore et al., 2007). Moreover, the cell surface adhesion molecule CD44 complexes with Ezrin to enhance tumor cell adhesion and invasion when co-localized in PCa-endothelial cells, facilitating tumor progression (Herrlich et al., 2000; Harrison et al., 2002). Conversely, Protein 4.1B—a structural domain protein incorporating Ezrin—acts as a negative modulator in PCa, impeding its progression and metastasis (Wong et al., 2007). In the realm of traditional Chinese medicine, the active component baicalin downregulates Ezrin in PCa cells and mice, restraining cell proliferation, inducing apoptosis, arresting the cell cycle, and reducing tumor dimensions. Depletion of Ezrin heightens baicalin’s inhibitory impact on PCa cell proliferation, underscoring the pivotal regulatory role of baicalin intervention and Ezrin levels in PCa advancement (Ma et al., 2020). Furthermore, Ezrin small molecule inhibitors like NSC 305787 and NSC 668394, initially employed in osteosarcoma treatment (Bulut et al., 2012), hold potential for PCa therapy by hindering Ezrin phosphorylation and its interaction with actin.

### 3.4 α-Actinin-4 (ACTN4)

ACTN4, a member of the spectrin gene superfamily, is a cytoskeletal protein crucially linked to cell motility, cancer invasion, and metastasis. During the transition from androgen-dependent to androgen-independent PCa, ACTN4 expression escalates, stimulating the transcription of genes associated with proliferation and metastasis (S. Park et al., 2020). Differentiating protein expression in the serum exosomes of patients with CRPC, Ishizuya noted a specific elevation solely in ACTN4 levels in CRPC patients compared to those undergoing ADT. Notably, downregulating ACTN4 expression using RNA interference significantly curtailed the proliferation and invasion of PCa cells (Ishizuya et al., 2020). Controlled by the upstream zinc finger protein CTCF, ACTN4 is upregulated through the lncRNA HOXA11-AS/miR-518b signaling pathway, fostering PCa proliferation and migration (Xing et al., 2020). These studies illustrate that ACTN4 upregulation propels the proliferation and motility of PCa cells; however, no effective and targeted drugs for ACTN4 currently exist.

## 4 Actin filament nucleation in PCa

### 4.1 Formin family proteins

Formins play a pivotal role in actin nucleation and filament assembly, crucial in various cellular functions such as cell division, migration, and adhesion. INF2, a member of the formin subfamily, emerges as a PCa-linked regulator, impacting tumor progression through diverse pathways (Zhao et al., 2022). Commonly mutated in primary PCa, the SPOP gene triggers aberrant polyubiquitination of INF2, disrupting INF2’s facilitation of mitochondrial fission and fostering PCa development (Jin et al., 2017). Investigating Formin subfamily member DAAM2 gene mutations in Androgen Insensitivity Syndrome, Knerr highlighted their potential contribution to AR inactivation and PCa advancement. DAAM2-mediated actin assembly drives AR clustering in response to dihydrotestosterone, inducing transcriptionally active droplet formation and potentially influencing PCa progression towards androgen deprivation resistance (Knerr et al., 2023). Silencing of Formin subfamily member DIAPH3 stimulates the formation and shedding of extracellular vesicles in PCa cells, hastening tumorigenesis and metastasis *in vivo* by modulating the tumor microenvironment (Kim et al., 2014). Notably, DIAPH3 inhibition of oncosome formation in EVs is counteracted by the loss of DIAPH3 gene expression in metastatic PCa cells (Di Vizio et al., 2009). Depletion of DIAPH3, as identified by Reis-Sobreiro et al., prompts nuclear instability and confers an amoeboid-like phenotype to PCa cells, fostering tumor cell propagation and migration (Morley et al., 2014; Reis-Sobreiro et al., 2018). In a contrasting effect, DIAPH3 knockdown or silencing heightens susceptibility to paclitaxel and epothilone B drugs (Morley et al., 2015; Lin and Windhorst, 2016). Thus, the intricate involvements of various Formin subfamily members in PCa progression, encompassing ubiquitination, mutations, and gene loss, position them as vital regulators and prospective therapeutic targets for impeding tumor cell proliferation and migration.

### 4.2 Actin-related protein 2/3 complex (Arp2/3 complex)

The Arp2/3 complex is a well-known actin nucleation protein consisting of ARP2, ARP3, and ARPC1 to ARPC5 (Goley and Welch, 2006). Together with ABPs like Formin, N-WASP, WAVE1, and Cortactin, the Arp2/3 complex collectively regulates the formation of secondary protrusions from primary protrusions (Yang and Svitkina, 2011; Giri et al., 2013). This process enables PCa cells to enhance their migration and invasion abilities by developing invasive pseudopods and degrading the extracellular matrix (Desai et al., 2008). Analysis of databases indicates that ARPC1A can independently predict lymph node metastasis and disease prognosis in PCa (N. Xu et al., 2020). Functional *in vitro* experiments have revealed that Signal Transducer and Activator of Transcription 3 (STAT3) plays a role in regulating the transcription of ARPC1A. Decreasing ARPC1A expression promotes ferroptosis, ultimately leading to reduced cell viability and invasion capabilities of PCa cells (Ji et al., 2022). Suppression of ARPC1B expression impedes the migration and invasion of PCa cells, causing cell cycle arrest in the G2/M phase. Assessing patients’ clinical prognosis can be achieved by examining the combination of ARPC1B with PTEN or ERG (Gamallat et al., 2022). Krüppel-like factor 4 (KLF4) acts as a transcriptional activator of ARPC5, promoting PCa progression through the activation and upregulation of ARPC5 via the Notch and Wnt pathways (Qu et al., 2023). The aforementioned studies provide initial insights into the significant roles of ARPC1A, ARPC1B, and ARPC5 within the Arp2/3 complex in PCa, underscoring their importance in cell migration and invasion. Targeting the activity of the Arp2/3 complex with specific drugs may present novel therapeutic approaches for PCa treatment.

### 4.3 WASP/WAVE family proteins

**FIGURE 5:**
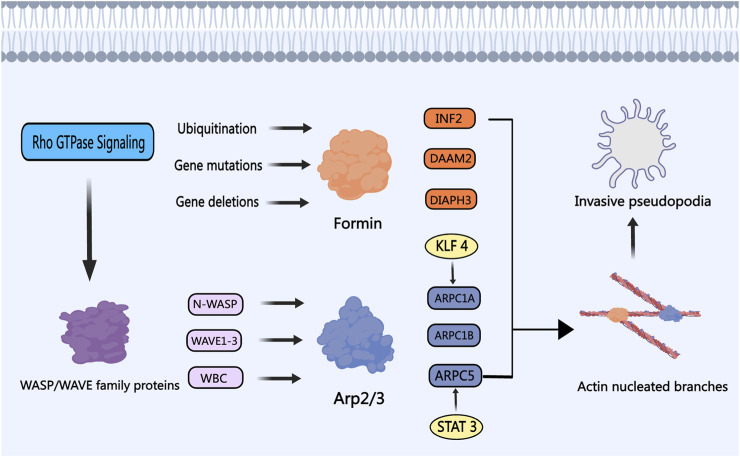
The Role of Nucleation Proteins in PCa. The WASP/WAVE family proteins are modulated by upstream signaling factors, particularly the Rho GTPase protein family, which in turn activates the nucleation activity of the Arp2/3 complex. Transcription activators like STAT3 and KLF4 enhance the Arp2/3 complex activity, fostering the development of invasive and metastatic phenotypes in PCa cells. Members of the Formin subfamily, including INF2, DAAM2, and DIAPH3, undergo ubiquitination, gene mutation, and gene loss in PCa, respectively. Depletion of Formins results in the development of invasive pseudopodia, thereby promoting PCa cell invasion and metastasis.

The Wiskott-Aldrich Syndrome Protein (WASP) and WASP Family Verprolin Homologous Protein (WAVE) family proteins, encompassing subtypes like WAVE1, WAVE2, WAVE3, WASP, and N-WASP, act as scaffold proteins in activating the ARP 2/3 complex (Takenawa and Suetsugu, 2007). Fernando and Moazzam have reported a strong correlation between WAVE1, WAVE3, and the invasiveness of metastatic PCa cells (Fernando et al., 2008; 2010; Moazzam et al., 2015). The WASP/WAVE proteins are considered potential therapeutic targets against PCa, with N-WASP believed to be a more potent activator of the ARP 2/3 complex compared to Cortactin (Burianek and Soderling, 2013; Mughees et al., 2021). These family proteins are regulated by upstream Rho GTPase protein signals, triggering ARP 2/3 complex activation, actin cytoskeleton remodeling, and the formation of invadopodia (Weeks et al., 2016; Rana et al., 2021). Hebbrecht has developed nanobodies that target N-WASP, modulating the N-WASP-Arp2/3 complex interaction, thereby reducing invasive pseudopod formation and matrix degradation during PCa invasion (Hebbrecht et al., 2017). Collapsin Response Mediator Protein-1 (CRMP1) acts as a tumor suppressor and exhibits reduced expression in advanced PCa tissues. CRMP1 has the capability to bind to WAVE1 and the ARP 2/3 complex, inhibiting EMT and the invasive potential of PCa cells (Cai et al., 2017). The WAVE Regulatory Complex (WRC), crucial for activating ARP 2/3, consists of proteins such as ABI2 and WAVE2. PIM1 kinase can phosphorylate ABI2, enhancing WRC activity and promoting actin dynamics to drive PCa invasion (Jensen et al., 2023). By targeting the pivotal role of the WASP/WAVE family proteins in actin nucleation branching, the development of specific inhibitors or antagonists holds promise. For instance, Peterson identified that cyclomodulin 187–1 selectively targets N-WASP, disrupting the interaction between WASP/WAVE family proteins and the ARP 2/3 complex, presenting a potential therapeutic strategy for PCa (Peterson et al., 2001). Figure 5 illustrates the roles and mechanisms of nucleation proteins in PCa.

## 5 Actin filament assembly and elongation in PCa

### 5.1 eEF1A

The eEF1A family proteins, comprising eEF1A1 and eEF1A2, play a role in regulating actin filament assembly in PCa progression. In both cultured PCa cells and tissues, eEF1A2 demonstrates elevated levels of mRNA and protein compared to eEF1A1 (Scaggiante et al., 2012). Conversely, eEF1A1 is notably expressed in osteoblasts neighboring metastatic tumor cells (Rehman et al., 2012). Enhanced eEF1A1 expression has been identified in PCa patients with Gleason scores of seven to eight, correlating positively with unfavorable clinical outcomes. Notably, the GT75 DNA aptamer developed by Bosutti et al. targets the eEF1A1-actin complex in invasive PCa cells, leading to reduced cell viability and increased cell death, suggesting eEF1A1 as a potential therapeutic target for advanced PCa (Bosutti et al., 2022). In a study screening PCa resistance-related genes to metformin, Chen et al. observed that metformin resistance in PCa is linked to eEF1A1 activation (Chen et al., 2020). Consequently, therapeutic strategies aiming to disrupt the interaction between eEF1A and actin may prove beneficial in inhibiting actin filament assembly and PCa cell migration.

### 5.2 Tropomyosin (TPM)

Tropomyosin encompasses two main subtypes, TPM1 and TPM2, which interact with actin to construct a stable cytoskeleton. In PCa cells, both TPM1 and TPM2, known as tumor suppressor genes, exhibit suppressed expression levels (Assinder et al., 2010). The reduced expression of TPM2 in PCa patient tumor tissues is significantly correlated with a less favorable prognosis, suggesting that downregulation of TPM2 predicts poor outcomes for PCa patients (Varisli, 2013). TPM2 inhibits androgen-independent proliferation, invasion, and the growth of subcutaneous xenograft tumors in PCa cells by activating the Hippo pathway and suppressing downstream target genes of YAP1 (Wu et al., 2023). Therefore, targeting the expression or functional modulation of TPM2 could be an effective approach to hinder PCa progression. Additionally, TPM1 expression is downregulated in PCa cells, potentially linked to exosomal miR-183. miR-183 enhances the proliferation and invasion of PCa cells by reducing TPM1 expression (Dai and Gao, 2021). Future strategies involving the upregulation of TPM or the activation of the Hippo pathway through gene editing technologies to exert its anti-cancer effects may hold promise as effective therapeutic avenues.

### 5.3 Vasodilatation stimulates phosphoprotein (VASP)

VASP, a member of the Ena/VASP protein family, plays a crucial role in initiating PCa migration through phosphorylation. Nitric oxide (NO) in prostate cells can activate the cGMP/PKG signaling pathway, resulting in VASP phosphorylation (Cook and Haynes, 2007). Lysophosphatidic acid (LPA), a bioactive lipid associated with motility and invasive properties in various cancer cell lines, induces VASP phosphorylation upon activation of LPA receptors, a pivotal step in lamellipodia formation and cell migration (Hasegawa et al., 2008). Microcystin-LR (MC-LR), a potential human carcinogen, promotes microfilament rearrangement and cell invasion in PCa cells by enhancing VASP, Ezrin, and ERK phosphorylation (X. Zhang et al., 2022). Given the significance of VASP phosphorylation in PCa migration, therapeutic strategies targeting molecules like NO, LPA, and MC-LR to inhibit or antagonize VASP phosphorylation, or directly block VASP phosphorylation, offer promising avenues for therapeutic interventions.

### 5.4 Profilin

**FIGURE 6:**
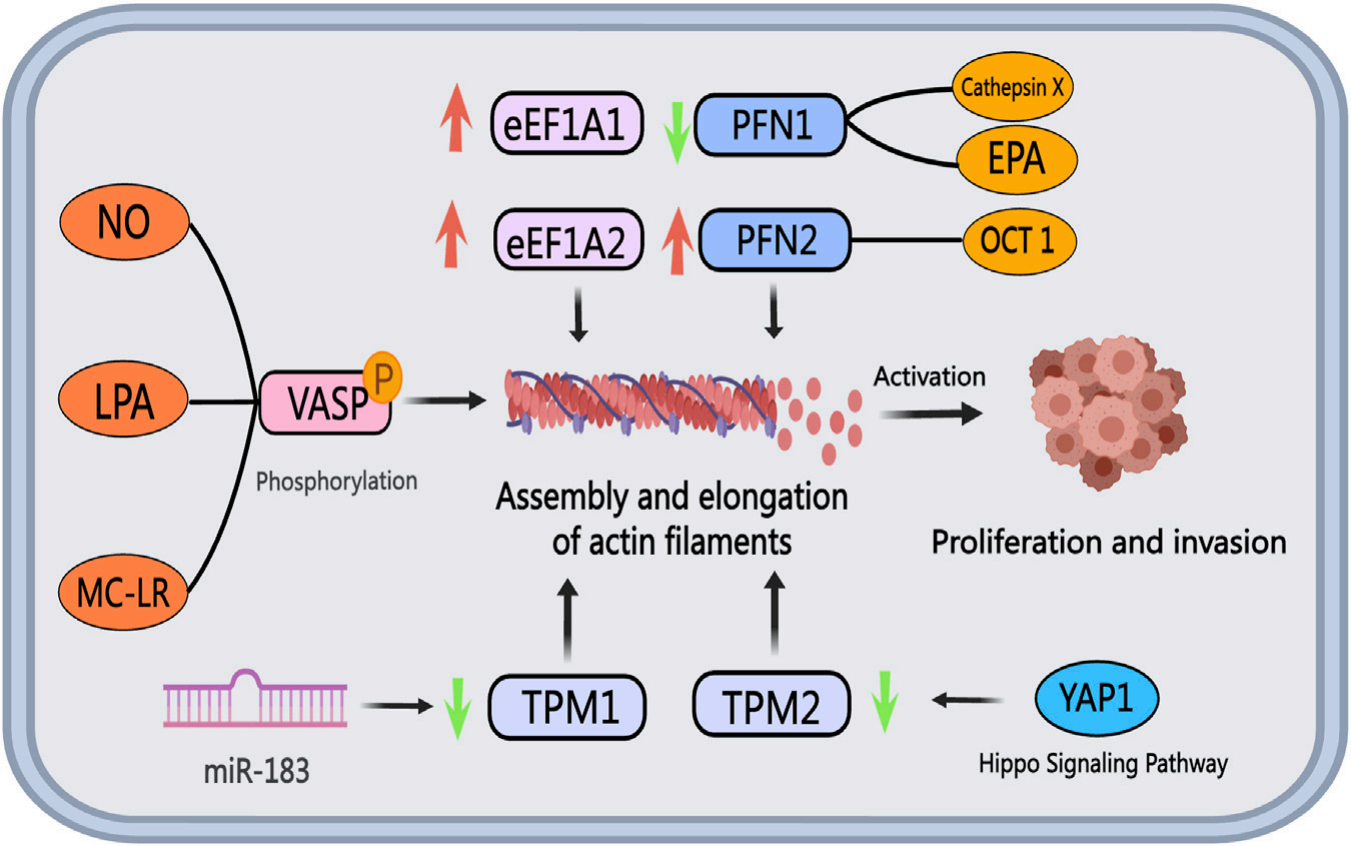
The role of actin-binding proteins in PCa. The eEF1A family proteins, eEF1A1, and eEF1A2, display upregulation in PCa cells and tissues. Tropomyosin, acting as a tumor suppressor gene, exhibits diminished expression levels of TPM1 and TPM2 in PCa, facilitating cancer cell invasion and metastasis. Extracellular vesicle miR-183 downregulates TPM1 expression, while reduced TPM2 expression impacts the Hippo pathway and downstream YAP1 target genes. NO, LPA, and MC-LR promote VASP phosphorylation, influencing cytoskeletal remodeling and fostering PCa invasion and metastasis by modulating actin filament assembly and elongation. Cathepsin X and EPA inhibit PFN1 expression, whereas OCT1 boosts PFN2 expression, resulting in increased tumor cell proliferation and invasion capacity.

Profilin, a widely distributed actin filament-binding protein with a molecular weight of around 15 kDa, plays a vital role in regulating actin filament polymerization and elongation dynamics along with cofilin (FU et al., 2008). These proteins collectively influence various processes in tumor progression, including migration and invasion of PCa cells. Typically, invasive tumors exhibit low Profilin expression, leading to mitotic defects, chromosomal instability, and heightened malignancy in tumor cells (Scotto et al., 2023). Current research in PCa predominantly focuses on Profilin subtypes PFN1 and PFN2. Studies indicate that increased PFN1 expression enhances the effectiveness of chemotherapy drugs and chemotherapy-induced cell death (Saurav and Manna, 2022). Cathepsin X, identified as a PCa promoter, may hinder clathrin binding by cleaving PFN1 (Pečar and Kos, 2015). The compromised function of PFN1 due to cathepsin X action results in elevated motility and invasiveness of PCa cells (Pečar et al., 2013). Eicosapentaenoic acid (EPA) reduces the acetylation level of PFN1, affecting its localization and impeding PCa cell migration and invasion by inhibiting lamellipodia or filopodia formation (C. He et al., 2024). Furthermore, through interaction with AR, the transcription factor OCT1 enhances signaling pathways linked to PCa progression, with PFN2 identified as a target gene of OCT1 in AR-negative PCa cells. Knockdown of PFN2 significantly inhibits tumor cell growth, proposing PFN2 targeting as a potential therapeutic avenue for CRPC (Obinata et al., 2022). Figure 6 illustrates the roles and mechanisms of ABPs in PCa.

## 6 Actin filament capping in PCa

### 6.1 Capping proteins

Capping proteins bind to the barbed ends of actin filaments, facilitating rapid filament growth and influencing cell motility. In PCa, CapG expression is notably elevated compared to matched non-cancerous prostate tissue, impacting cell proliferation and apoptosis through the activation of the Caspase 6/Caspase 9/Bcl-2/p-Akt/Akt signaling pathway (T. Li et al., 2016). The heterodimeric form of CapZ, consisting of CAPZA1 and CAPZB2, acts as a substrate for the oncogenic PIM1 kinase. Phosphorylation of CAPZ by PIM1 diminishes its actin-capping function, enhancing PCa cell adhesion and migration (Santio et al., 2020). Targeting the increased expression and phosphorylation of capping proteins in PCa presents a potential therapeutic approach.

### 6.2 Gelsolin (GSN)

Gelsolin, a member of the gelsolin superfamily, primarily functions in severing and capping actin filaments, thereby regulating the cell cytoskeleton. Acting as an AR co-activator, GSN exhibits high expression in late-stage PCa resistant to endocrine therapy (Culig et al., 2005). Oelrich et al. successfully inhibited the IL6-mediated neuroendocrine differentiation in NEPC cells by targeting and reducing GSN expression (Oelrich et al., 2021). NEPC cells secrete neurotensin, which enhances PCa cell invasiveness by activating neurotensin receptors and fostering GSN-related processes (Hashimoto et al., 2015). Therefore, GSN plays a critical role in regulating the neuroendocrine differentiation in PCa. Targeting the interplay between GSN and AR holds promise as a potential therapeutic strategy for PCa (Nishimura et al., 2003).

## 7 Actin binding in PCa

Transgelin (TAGLN) is a protein that modulates actin polymerization, crucial for stabilizing and gelating the actin cytoskeleton. Proteomic and bioinformatic analyses indicate that TAGLN can be a valuable diagnostic biomarker for benign prostatic hyperplasia and PCa (Kmeťová Sivoňová et al., 2021; Su et al., 2023). TAGLN is often linked to tumor suppression, with reports of its downregulation in PCa tissue (Prasad et al., 2010). The decrease in TAGLN expression disrupts the actin cytoskeleton, a pivotal factor in PCa progression. TRAF6-mediated proteasomal degradation contributes to TAGLN downregulation, enhancing PCa cell proliferation and suppressing migration by activating the NF-κB and Myc signaling pathways (Wen et al., 2021). However, conflicting results from various studies on TAGLN and its cellular detection exist. Factors like TGF-β1 and kallikrein-related peptidase-4 (KLK4) induce CAF-like behaviors, increasing TAGLN expression in prostate stromal cell lines, thereby triggering extracellular matrix remodeling and invasive metastasis (Untergasser et al., 2005; Kryza et al., 2017). These findings highlight the differential expression of TAGLN in PCa tissues and the specific mechanisms involving regulatory targets like TRAF6, NF-κB, and Myc. In essence, gene regulation of TAGLN or interventions in upstream and downstream signaling targets may aid in restoring actin cytoskeleton stability, thus impeding PCa progression. The associated ABPs in PCa and their family members are detailed in Table 2.

**TABLE 2:** Different types of ABPs and their family members.

Types	Basic function	ABPs	Expression level in PCa
Severing protein	Severs Actin filament	Cofilin	High expression of dephosphorylated cofilin
Cross-linking protein	Cross-linked actin filaments	FlnA	Low expression of nuclear FlnA and high expression of AR/FlnA complex
		FSCN1	High expression of FSCN1
		Ezrin	High expression of phosphorylation Ezrin
		ACTN4	High expression of ACTN4
Nucleating protein	Nucleation to initiate actin polymerization	Formin	INF2 ubiquitination, DAAM2 gene mutation, and DIAPH3 gene loss
		Arp2/3 complex	High expression of ARPC1A, ARPC1B, and ARPC5
		WASP/WAVE	High expression of WAVE1-3, N-WASP, and WBC
Actin-filament-binding protein	Polymerizes and bound to Actin filament	eEF1A	High expression of eEF1A1 and eEF1A2
		TPM	Low Expression of TPM1 and TPM2
		VASP	High expression of phosphorylation VASP
		Profilin	Low expression of PFN1 and high expression of PFN2
Capping protein	Caps Actin filament to inhibit actin polymerization	Capping protein	High expression of CapG and phosphorylation of CapZ
		GSN	High expression of GSN
Actin-binding protein	Polymerizes and bound to actin	TAGLN	High or low expression of TAGLN

## 8 Discussion and prospect

PCa stands as one of the most prevalent malignant diseases among men globally, posing significant challenges like androgen deprivation resistance and bone metastasis in advanced treatment stages. These challenges intricately tie back to the roles of ABPs in governing the actin cytoskeleton. Recent research illustrates that ABPs not only impact the cytoskeleton stability but also regulate androgen signaling pathways, thus modulating the migration, invasion, and proliferation of PCa cells. It’s crucial to note that ABPs exhibit diverse functions rather than singular roles. Various ABPs likely play distinct parts in the pathogenesis and progression of PCa. For instance, cofilin acts in both severing actin filaments and governing actin polymerization and depolymerization. This article summarizes 14 documented ABPs linked to PCa, laying a foundation for conceptualizing subsequent PCa treatment strategies.

Nevertheless, this study primarily focuses on known ABPs associated with PCa, with still limited comprehensive understanding regarding the specific mechanisms of interaction between ABPs and the actin cytoskeleton. Furthermore, the molecular mechanisms of other ABPs not yet reported in PCa remain obscure, which might constrain our holistic comprehension of PCa pathogenesis. While some ABPs related to PCa have been preliminarily explored, numerous unanswered questions persist. Future investigations should delve deeper into the interplays between ABPs and the actin cytoskeleton, elucidating their precise roles in PCa pathogenesis and metastasis. Such in-depth insights will facilitate the development of targeted treatment approaches directed at ABPs to more effectively combat the existing challenges in PCa management.
